# Longitudinal Increases in Time to Surgery for Patients with Breast Cancer: A National Cohort Study

**DOI:** 10.1245/s10434-024-15723-w

**Published:** 2024-07-13

**Authors:** Gabriella N. Tortorello, Neha Shafique, Luke Keele, Carolyn G. Susman, Anushka Dheer, Oluwadamilola M. Fayanju, Julia Tchou, John T. Miura, Giorgos C. Karakousis

**Affiliations:** 1 Department of Surgery, Hospital of the University of Pennsylvania, Philadelphia, PA USA; 2 Perelman School of Medicine, University of Pennsylvania, Philadelphia, PA USA

**Keywords:** Time to surgery, Time to treatment, Breast cancer

## Abstract

**Background:**

Longer time to surgery (TTS) is associated with worse survival in patients with breast cancer. Whether this association has encouraged more prompt care delivery remains unknown.

**Methods:**

The National Cancer Database was used to identify patients ≥18 years of age diagnosed with clinical stage 0–III breast cancer between 2006 and 2019 for whom surgery was the first mode of treatment. A linear-by-linear test for trend assessed median TTS across the interval. Adjusted linear regression modeling was used to examine TTS trends across patient subgroups.

**Results:**

Overall, 1,435,584 patients met the inclusion criteria. The median age was 63 years (interquartile range [IQR] 53–72), 84.3% of patients were White, 91.1% were non-Hispanic, and 99.2% were female. The median TTS in 2006 was 26 days (IQR 16–39) versus 39 days in 2019 (IQR 27–56) [*p* < 0.001]. In a multivariable linear regression model, TTS increased significantly, with an annual increase of 0.83 days (95% confidence interval 0.82–0.85; *p* < 0.001). A consistent, significant increase in TTS was observed on subgroup analyses by surgery type, reconstruction, patient race, hospital type, and disease stage. Black race, Hispanic ethnicity, and having either Medicaid or being uninsured were significantly associated with prolonged TTS, as were mastectomy and reconstructive surgery.

**Conclusions:**

Despite evidence that longer TTS is associated with poorer outcomes in patients with breast cancer, TTS has steadily increased, which may be particularly detrimental to marginalized patients. Further studies are needed to ensure the delivery of timely care to all patients.

Delayed time to surgery (TTS) is associated with higher mortality in patients with breast cancer.^1–12^ Prior studies have explored the impact of various specific time intervals on patient outcomes, both to show a dose-effect proof of concept (longer delays lead to worse outcomes) and to establish a specific target for providing timely care in response to the question of how long is too long. A recent study identified 8 weeks as the TTS length after which patients begin to have worsened survival outcomes.^1^ Earlier work similarly found that breast cancer-related mortality increases for patients waiting 60 days or longer for surgical management as first course of treatment for their disease.^13^ Still, despite the strong body of evidence that timely surgery improves patient outcomes, little is known about whether this knowledge has affected care. In other words, are patients getting to the operating room more quickly? In this study, we sought to investigate longitudinal trends in TTS for breast cancer in the United States (US), as well as differences in TTS trends by patient-, disease-, treatment-, and hospital-level factors. Additionally, we sought to identify any variables associated with TTS of 8 weeks or longer and to characterize the proportion of patients with clinically significant delays in care.

## Methods

This was a retrospective cohort study using the National Cancer Database (NCBD), which includes patient data from more than 1500 medical centers accredited by the American College of Surgeons Commission on Cancer (CoC) and captures nearly 80% of all new cancer diagnoses in the US on an annual basis.^14^ We included all patients aged ≥18 years who were diagnosed with American Joint Committee on Cancer (AJCC) 8th Edition clinical stage 0–III breast cancer between 2006 and 2019 for whom surgery was the first mode of treatment. We excluded patients diagnosed in 2020 from our analysis because prior studies have suggested instability of NCDB data from this year, presumably in the context of the coronavirus disease 2019 (COVID-19) pandemic.^15^ We also excluded any patients for whom treatment course was unknown and any patients for whom TTS from diagnosis was listed as 0 days, as these patients were most likely diagnosed on excisional biopsy and were not representative of the more common care pathway we aimed to explore. Throughout the analysis, TTS was defined as days from diagnosis to definitive surgical management unless otherwise specified. Patients with missing data with respect to TTS and other factors included in our multivariable models were excluded from the analysis. Descriptive statistics were used to examine the overall characteristics of the study cohort. For continuous variables, we reported medians and interquartile ranges (IQRs) as the data were non-parametrically distributed; for categorical variables, we reported percentages. Statistical significance was set at a standard *p*-value <0.05, and all analyses were performed using Stata statistical software, version 17.0 (StataCorp LLC, College Station, TX, USA).

### Trends in Time to Surgery and Overall Survival

To examine the trends in TTS, we first calculated the median TTS for each year of diagnosis. We then conducted a linear-by-linear test for trend to determine the significance of observed changes over time. We also developed an adjusted linear regression model to predict TTS, in which year of diagnosis was considered the primary exposure variable. In addition, we conducted subgroup analyses according to disease stage, breast surgery type (mastectomy vs. lumpectomy), reconstruction status, patient race, and hospital type. Because some patients undergo multiple surgical procedures, we also examined trends in days from diagnosis to first surgical procedure, with the assumption that this value would be the same as TTS as defined as days to definitive surgical procedure for most patients. To examine overall survival (OS) in our study cohort, we examined disease stage-stratified OS by year of diagnosis using the Kaplan–Meier method.

### Predictors of Delays to Surgery

We created a binary variable of TTS of ≤8 weeks (LT8) or >8 weeks (GT8). We first compared patients in the LT8 and GT8 cohorts using Pearson’s Chi-square tests for categorical variables and Wilcoxon rank-sum tests for continuous variables. We then built a logistic regression model that considered the impact of patient, disease, and treatment factors on TTS. We selected factors that we hypothesized a priori to be significantly associated with the likelihood of delays to surgical care, and as we hypothesized that there may be a significant interaction between patient race and reconstruction status, we created an interaction term to test in the full model. We used backward stepwise selection to select the most significant variables for inclusion in the final logistic regression model.

## Results

### Demographics of the Cohort

Overall, 1,435,584 patients met the inclusion criteria. The median age was 63 years (IQR 53–72), 84.3% of patients were White, 91.1% were non-Hispanic, and 99.2% were female. The complete characteristics of the cohort are presented in Table 1. Across the study interval, there was an increase in the percentage of patients undergoing lumpectomy versus mastectomy (62.1% in 2006 vs. 67.0% in 2019; *p* < 0.001) and in those undergoing reconstruction among mastectomy patients (21.1% in 2006 vs. 46.1% in 2019; *p* < 0.001). There were no clinically significant changes in patient demographics, hospital factors, or disease stage at presentation over time. A total of 241,682 patients (16.8%) had TTS of GT8, whereas 1,193,902 (83.2%) were in the LT8 group. In 2006, only 10.1% of the patients had TTS GT8, and this number more than doubled to 23.8% in 2019.

**Table 1:** Characterstics of the cohort by time to surgery

		≤8 weeks	>8 weeks	*p*-Value
Age, years	<65	636,586 (53.3)	145,823 (60.3)	<0.001
	≥65	557,316 (46.7)	95,859 (39.7)	
Race	White	1,023,835 (85.8)	186,297 (77.1)	<0.001
	Black	107,665 (9.0)	37,852 (15.7)	
	AAPI	44,512 (3.7)	12,373 (5.1)	
	Other	17,890 (1.5)	5160 (2.1)	
Ethnicity	Non-Hispanic	1,094,536 (91.7)	213,788 (88.5)	<0.001
	Hispanic	52,474 (4.4)	20,989 (8.7)	
	Other	46,892 (3.9)	6905 (2.9)	
CDCC score	0	976,860 (81.8)	195,130 (80.7)	<0.001
	1	166,758 (14.0)	34,368 (14.2)	
	2	35,477 (3.0)	8009 (3.3)	
	3+	14,807 (1.2)	4175 (1.7)	
Insurance status	Uninsured	14,478 (1.2)	5434 (2.2)	<0.001
	Privately insured	596,445 (50.0)	119,693 (49.5)	
	Medicaid	50,101 (4.2)	20,696 (8.6)	
	Medicare	508,822 (42.6)	88,939 (36.8)	
	Unknown	24,056 (2.0)	6920 (2.9)	
Clinical disease stage	0	227,745 (19.1)	65,824 (27.2)	<0.001
	1	643,970 (53.9)	116,598 (48.2)	
	2	263,243 (22.0)	47,366 (19.6)	
	3	58,944 (4.9)	11,894 (4.9)	
Surgery type	Lumpectomy	773,718 (64.8)	115,604 (47.8)	<0.001
	Mastectomy	420,184 (35.2)	126,078 (52.2)	
Reconstruction status	None	1,049,549 (87.9)	174,730 (72.3)	<0.001
	Implant-based	88,824 (7.4)	40,464 (16.7)	
	Tissue-based/combined	55,529 (4.7)	26,488 (11.0)	
Hospital type	Non-academic	847,935 (73.0)	144,794 (62.0)	<0.001
	Academic	313,191 (27.0)	88,620 (38.0)	
Hospital setting	Metropolitan	1,017,263 (87.2)	213,490 (90.7)	<0.001
	Urban	131,873 (11.3)	19,749 (8.4)	
	Rural	17,251 (1.5)	2240 (1.0)	

Data are expressed as *n* (%)

*AAPI* Asian American/Pacific Islander, *CDCC* Charlson–Deyo Comorbidity Class

### Trends in Time to Surgery

**Fig. 1:**
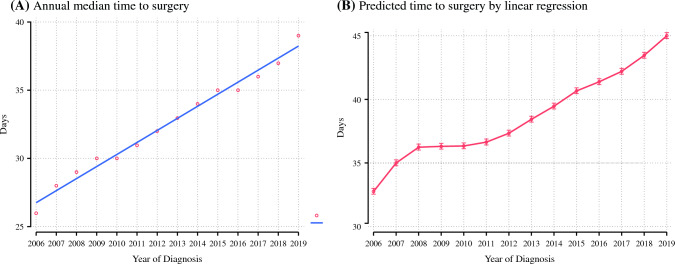
**a** Median time to surgery by year of diagnosis. **b** Predicted annual time to surgery on linear regression with 95% confidence intervals

The overall median TTS in 2006 was 26 days (IQR 16–39), while in 2019, the median TTS was 39 days (IQR 27–56) [Kruskal–Wallis *p* < 0.001]. A linear-by-linear test for trend showed a statistically significant increase in the median TTS over the study period (*p* = 0.003). Figure 1a shows the median TTS based on year of diagnosis. In a multivariable linear regression model using year of diagnosis as a primary, ordinal exposure variable, and adjusting for patient, disease, and treatment factors, TTS increased significantly over the period, with an average increase of 0.83 days per year (95% confidence interval [CI] 0.82–0.85; *p* < 0.001). The annual TTS predicted using this model is shown in Fig. 1b. When considering time from diagnosis to first surgical procedure, we observed a similar trend, with median time to first surgery in 2006 of 25 days compared with 39 days in 2019 (*p* < 0.001).

**Fig. 2:**
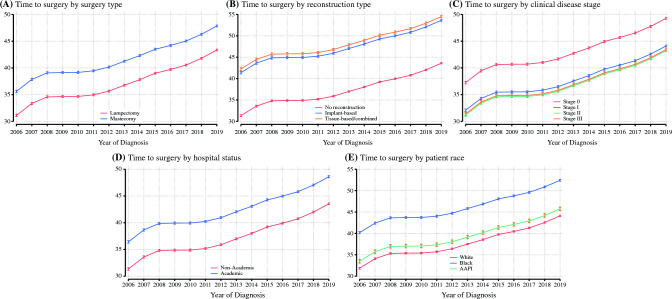
Subgroup analyses of predicted time to surgery in days by multivariable linear regression with 95% confidence intervals by: **a** surgery type (lumpectomy vs. mastectomy); **b** reconstruction status; **c** disease stage; **d** hospital academic status; and **e** patient race

We observed previously described disparities in TTS by surgery type, reconstruction status, patient race and ethnicity, disease stage, and hospital setting.^2,16^ For example, White patients had a median TTS of 33 days overall (IQR 22–47), while for Black patients, TTS was 39 days (IQR 24–54) [Kruskal–Wallis *p* < 0.001]. Lumpectomy was associated with a median TTS of 31 days (IQR 21–44) versus mastectomy of 38 days (IQR 25–55) [Kruskal–Wallis *p* < 0.001]. Reconstruction status had a particularly profound impact, with patients who did not undergo reconstruction having an overall median TTS of 31 days (IQR 21–45) compared with implant-based reconstruction TTS of 45 days and tissue-based or combined tissue/implant-based reconstruction overall TTS of 46 days (Kruskal–Wallis; *p* < 0.001). Patients who received surgical care at the reporting facility had a median TTS of 33 days (IQR 22–49), while patients who received surgical care elsewhere had an overall median TTS of 32 days (IQR 21–47) [Kruskal–Wallis *p* < 0.001]. No cohort was spared the impact of worsening delays in surgical care, and a consistent and significant increase in TTS was observed in subgroup analyses by each of these factors, as shown in Fig. 2.

### Predictors of Time to Surgery Greater Than 8 Weeks

On multivariable analysis adjusted for patient, disease, and treatment factors, patients aged 65 years and older had 7% decreased odds of TTS GT8 (odds ratio [OR] 0.93, 95% CI 0.91–0.94; *p* < 0.001). Black patients had 82% increased odds of TTS GT8 compared with White patients (OR 1.82, 95% CI 1.80–1.85; *p* < 0.001), and patients who were Asian or Pacific Islander had 18% increased odds (OR 1.18, 95% CI 1.15–1.21; *p* < 0.001). Patients who were Hispanic also had greater odds of delays in surgical care compared with non-Hispanic patients (OR 1.70, 95% CI 1.66–1.73; *p* < 0.001). Having a greater number of medical comorbidities, as measured by the Charlson–Deyo combined comorbidity score, was associated with increased odds of TTS GT8 (CDCC score 3 vs. 0: OR 1.53, 95% CI 1.47–1.58; *p* < 0.001). Patients who underwent either type of reconstruction had 2.2 times the odds of TTS GT8 (OR 2.19, 95% CI 2.16–2.22; *p* < 0.001), and patients who underwent mastectomy had 57% increased odds of TTS GT8 compared with lumpectomy patients (OR 1.57, 95% CI 1.55–1.59; *p* < 0.001). We found no significant interaction between patient race and reconstruction status; therefore, this term was excluded from our final model. The results of our multivariable logistic regression model are presented in Table 2.

**Table 2:** Factors associated with TTS >8 weeks as predicted by multivariable logistic regression model

		OR	95% CI	*p*-value
Age, years	<65	1 (reference)		
	≥65	0.93	0.91–0.94	<0.001
Race	White	1 (reference)		
	Black	1.82	1.80–1.85	<0.001
	AAPI	1.18	1.15–1.21	<0.001
	Other	1.27	1.22–1.32	<0.001
Ethnicity	Non-Hispanic	1 (reference)		
	Hispanic	1.70	1.66–1.73	<0.001
	Other	0.83	0.81–0.85	<0.001
CDCC score	0	1 (reference)		
	1	1.07	1.05–1.08	<0.001
	2	1.23	1.19–1.26	<0.001
	3+	1.53	1.47–1.58	<0.001
Insurance status	Uninsured	1 (reference)		
	Privately insured	0.55	0.53–0.57	<0.001
	Medicaid	1.06	1.02–1.11	<0.001
	Medicare	0.65	0.63–0.68	<0.001
	Unknown	0.82	0.78–0.86	<0.001
Clinical disease stage	0	1 (reference)		
	1	0.67	0.66–0.68	<0.001
	2	0.57	0.56–0.58	<0.001
	3	0.56	0.55–0.58	<0.001
Surgery type	Lumpectomy	1 (reference)		
	Mastectomy	1.57	1.55–1.59	<0.001
Reconstruction status	None	1 (reference)		
	Implant-based	2.17	2.14–2.21	<0.001
	Tissue-based/combined	2.25	2.20–2.29	<0.001
Hospital type	Non-academic	1 (reference)		
	Academic	1.49	1.47–1.50	<0.001
Hospital setting	Metropolitan	1 (reference)		
	Urban	0.82	0.80–0.83	<0.001
	Rural	0.70	0.67–0.73	<0.001

*TTS* Time to surgery, *OR* Odds ratio, *CI* Confidence interval, *AAPI* Asian American/Pacific Islander

### Trends in Overall Survival

**Fig. 3:**
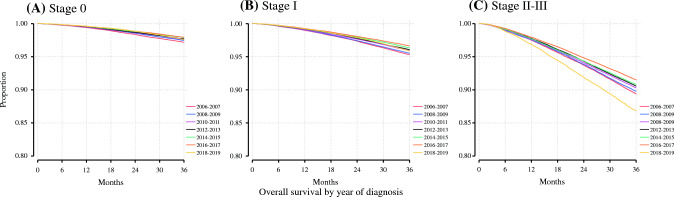
AJCC clinical stage-stratified overall survival by year of diagnosis. **a** Stage 0; **b** stage I; **c** stage II–III. *AJCC* American Joint Committee on Cancer

Across the study period, we observed a general trend toward improved OS by Kaplan–Meier analysis (Fig. 3). In 2006–2007, the 3-year OS for stage II–III patients in our study cohort was 89.4% (95% CI 89.1–89.7%), while the 3-year OS for stage II–III patients diagnosed in 2016–2017 was 91.5% (95% CI 91.4–91.7%) [*p* < 0.001]. Notably, for patients diagnosed in 2018 and 2019, for whom follow-up was predominantly observed during the COVID-19 pandemic, this trend toward improved OS did not persist.

## Discussion

A growing body of literature suggests that delays in surgical care of breast cancer are associated with increased mortality.^1–12^ In the present study, examining TTS in a national cohort between 2006 and 2019, we observed a significant annual increase in TTS across the entire study period. Although certain factors were found to be associated with surgical delays in any given year, each patient-, treatment-, and disease-specific subgroup experienced the same trends with longer delays over time. Furthermore, the rate of increase in TTS was generally consistent regardless of surgical type, reconstruction status, disease stage, patient demographic factors, or hospital type.

Accordingly, with each passing year of our study, an increased proportion of patients waited longer than 8 weeks for surgery. This specific time interval has recently been suggested as an important benchmark after which OS worsens, and defining TTS as a binary variable may allow for clearer quality metrics by which hospital systems and providers may be adjudicated.^1^ In fact, the CoC recently developed a new quality metric to be reported in the NCDB assessing whether patients with AJCC clinical stage I–III breast cancer underwent “first therapeutic surgery in a non-neoadjuvant setting” within 60 days of diagnosis.^17^

In the current study, we found that reconstruction, mastectomy, non-White race, Hispanic ethnicity, greater number of comorbidities, and lack of health insurance were all associated with increased odds of waiting more than 8 weeks for primary surgical management of breast cancer. On the other hand, being diagnosed at a higher disease stage was associated with decreased odds of delays to care. These findings are consistent with care disparities previously described in the medical literature.^1–3,5,16,18^ As TTS increases overall, a larger percentage of patients are waiting for more than 8 weeks and may be at risk of dying due to delays in care, and those patients who were already at risk for longer TTS may be the most vulnerable. It is important to note that there has been a steady improvement in breast cancer mortality over the past two decades, in part due to significant advances in adjuvant therapies.^19^ We observed this same trend in our study cohort with improved OS over time prior to 2018–2019, at which time we hypothesize that death due to COVID-19 was a significant competing risk. Still, longer delays in surgical care may represent an opportunity to further improve outcomes over and above what has already been accomplished. Furthermore, we know that significant outcome disparities exist, and Black patients continue to experience 40% higher mortality rates than White patients.^19^ Mitigating delays in care could be particularly impactful in this population and lead to a more pronounced reduction in mortality than already observed. Furthermore, the rising TTS may have a significant impact beyond patient survival, including increasingly burdensome healthcare system costs, as well as patient stress and emotional well-being.

This study examines more current data than previously published.^2,18,20^ One prior study compared TTS between 1992 and 2005 and found longer wait times in the latter year but did not look specifically at time trends over the entire interval.^2^ Another study describing trends in TTS between 2003 and 2011 found an increase in TTS,^18^ although these data also predate the strongest evidence we have that delays in surgical care significantly impact mortality, after which we may have hoped and expected to see a reversal in trends.^1,3–6,8,9,13^

The exact cause of delays in surgical care is unknown and likely multifactorial. As previous authors have suggested, there is an interplay between patient-, provider-, and system-level delays that may each be contributing.^13,20–22^ Patients might have greater access to breast cancer education and thereby be more inclined to seek second opinions that could unwittingly impact timely treatment.^23^ Notably, we found that patients who received surgical care away from the diagnosing facility did not face increased delays as hypothesized but in fact waited, on average, one less day for surgery, although the clinical significance of this finding is likely limited. Multidisciplinary cancer care can contribute to increased time to treatment if not optimally coordinated.^24^ For example, breast reconstruction requires meeting with both a surgical oncologist and plastic surgeon and finding a mutual schedule and operating room availability.^25^ Still, prioritization of short TTS should not outweigh the vital importance of multidisciplinary care, including upfront involvement of medical oncology and genetics, which has been well-established as improving patient outcomes.^26–30^ While prior literature has suggested convincing hypotheses as to why delays happen at any time point, we have a less clear understanding as to why the problem is getting worse. In addition, although we identified many factors statistically associated with longer TTS, the clinical significance of these findings varies, and those factors that are most targetable for quality improvement implementation may be the most vital.

Although our study provides valuable insights into recent trends in TTS for patients with breast cancer, there are a few noteworthy limitations. First, the NCDB includes only information from CoC-accredited institutions; thus, its use might introduce selection bias, as these institutions do not fully represent the diversity of healthcare settings across the US. In addition, we excluded patients with missing data based on the assumption that data are missing at random, when there may be factors associated with missingness that are unevenly introduced across study years, thereby introducing bias into the study and limiting the generalizability of the findings. Furthermore, there may be important unmeasured confounders, including patient-level income and education levels, that we were unable to adjust for in our analyses. Furthermore, in performing a large, national, retrospective study, we were unable to explore patient or provider perspectives or experiences, which could provide a more comprehensive and nuanced understanding of delays in surgical care. Our study, which is largely descriptive, cannot tell us what specific factors contribute most to surgical delays, although the reported findings may better inform future causal analyses and qualitative work.

## Conclusion

Despite these limitations, our results suggest that TTS is increasing in patients who are newly diagnosed with breast cancer and who are undergoing surgical resection as initial treatment. Although the full clinical implications of this trend remain unclear, no single patient group appears to be immune. Nearly 300,000 women in the US will be diagnosed with breast cancer this year alone, and small annual increases in TTS may translate to many potentially mitigable patient deaths, even as overall breast cancer-related mortality improves.^31^ Further studies are critically important to explore modifiable individual or system-level contributors to delayed treatment for patients diagnosed with this common malignancy.

## References

[1] Wiener AA, Hanlon BM, Schumacher JR, Vande Walle KA, Wilke LG, Neuman HB. Reexamining time from breast cancer diagnosis to primary breast surgery. *JAMA Surg*. 2023;158(5):485–92. 10.1001/jamasurg.2022.8388. 36857045 10.1001/jamasurg.2022.8388 PMC9979003

[2] Bleicher RJ, Ruth K, Sigurdson ER, et al. Preoperative delays in the US Medicare population with breast cancer. *J Clin Oncol*. 2012;30(36):4485–92. 10.1200/JCO.2012.41.7972. 23169513 10.1200/JCO.2012.41.7972 PMC3518727

[3] Prakash I, Thomas SM, Greenup RA, et al. Time to surgery among women treated with neoadjuvant systemic therapy and upfront surgery for breast cancer. *Breast Cancer Res Treat*. 2021;186(2):535–50. 10.1007/s10549-020-06012-7. 33206290 10.1007/s10549-020-06012-7 PMC7994184

[4] Eaglehouse YL, Georg MW, Shriver CD, Zhu K. Time-to-surgery and overall survival after breast cancer diagnosis in a universal health system. *Breast Cancer Res Treat*. 2019;178(2):441–50. 10.1007/s10549-019-05404-8. 31414244 10.1007/s10549-019-05404-8

[5] Smith EC, Ziogas A, Anton-Culver H. Delay in surgical treatment and survival after breast cancer diagnosis in young women by race/ethnicity. *JAMA Surg*. 2013;148(6):516–23. 10.1001/jamasurg.2013.1680. 23615681 10.1001/jamasurg.2013.1680

[6] Mateo AM, Mazor AM, Obeid E, et al. Time to surgery and the impact of delay in the non-neoadjuvant setting on triple-negative breast cancers and other phenotypes. *Ann Surg Oncol*. 2020;27(5):1679–92. 10.1245/s10434-019-08050-y. 31712923 10.1245/s10434-019-08050-y PMC7145740

[7] Zhu S, Li S, Huang J, Fei X, Shen K, Chen X. Time interval between breast cancer diagnosis and surgery is associated with disease outcome. *Sci Rep*. 2023;13(1):12091. 10.1038/s41598-023-39259-3. 37495705 10.1038/s41598-023-39259-3 PMC10372101

[8] Eriksson L, Bergh J, Humphreys K, Warnberg F, Tornberg S, Czene K. Time from breast cancer diagnosis to therapeutic surgery and breast cancer prognosis: A population-based cohort study. *Int J Cancer*. 2018;143(5):1093–104. 10.1002/ijc.31411. 29603736 10.1002/ijc.31411

[9] McLaughlin JM, Anderson RT, Ferketich AK, Seiber EE, Balkrishnan R, Paskett ED. Effect on survival of longer intervals between confirmed diagnosis and treatment initiation among low-income women with breast cancer. *J Clin Oncol*. 2012;30(36):4493–500. 10.1200/JCO.2012.39.7695. 23169521 10.1200/JCO.2012.39.7695 PMC3518728

[10] Shin DW, Cho J, Kim SY, et al. Delay to curative surgery greater than 12 weeks is associated with increased mortality in patients with colorectal and breast cancer but not lung or thyroid cancer. *Ann Surg Oncol*. 2013;20(8):2468–76. 10.1245/s10434-013-2957-y. 23529782 10.1245/s10434-013-2957-y

[11] Khorana AA, Tullio K, Elson P, et al. Time to initial cancer treatment in the United States and association with survival over time: An observational study. *PLoS One*. 2019;14(3):e0213209. 10.1371/journal.pone.0213209. 30822350 10.1371/journal.pone.0213209 PMC6396925

[12] Richards MA, Westcombe AM, Love SB, Littlejohns P, Ramirez AJ. Influence of delay on survival in patients with breast cancer: a systematic review. *Lancet*. 1999;353(9159):1119–26. 10.1016/s0140-6736(99)02143-1. 10209974 10.1016/s0140-6736(99)02143-1

[13] Bleicher RJ, Ruth K, Sigurdson ER, et al. Time to surgery and breast cancer survival in the United States. *JAMA Oncol*. 2016;2(3):330–9. 10.1001/jamaoncol.2015.4508. 26659430 10.1001/jamaoncol.2015.4508 PMC4788555

[14] American College of Surgeons. National Cancer Database.

[15] Lum SS, Browner AE, Palis B, et al. Disruption of national cancer database data models in the first year of the COVID-19 pandemic. *JAMA Surg*. 2023;158(6):643–50. 10.1001/jamasurg.2023.0652. 37043215 10.1001/jamasurg.2023.0652

[16] Reeder-Hayes KE, Mayer SE, Olshan AF, et al. Race and delays in breast cancer treatment across the care continuum in the Carolina Breast Cancer Study. *Cancer*. 2019;125(22):3985–92. 10.1002/cncr.32378. 31398265 10.1002/cncr.32378 PMC6819218

[17] American College of Surgeons. NCDB Quality Measure Improvements Announced. Available at: https://www.facs.org/quality-programs/cancer-programs/national-cancer-database/quality-of-care-measures/#:~:text=For%20patients%20with%20AJCC%20Clinical,including%2060%20days%20of%20diagnosis. Accessed 23 Apr 2024.

[18] Liederbach E, Sisco M, Wang C, et al. Wait times for breast surgical operations, 2003–2011: A report from the National Cancer Data Base. *Ann Surg Oncol*. 2015;22(3):899–907. 10.1245/s10434-014-4086-7. 25234018 10.1245/s10434-014-4086-7

[19] Giaquinto AN, Sung H, Miller KD, et al. Breast cancer statistics, 2022. *CA Cancer J Clin*. 2022;72(6):524–41. 10.3322/caac.21754. 36190501 10.3322/caac.21754

[20] Bilimoria KY, Ko CY, Tomlinson JS, et al. Wait times for cancer surgery in the United States: Trends and predictors of delays. *Ann Surg*. 2011;253(4):779–85. 10.1097/SLA.0b013e318211cc0f. 21475020 10.1097/SLA.0b013e318211cc0f

[21] Bairati I, Fillion L, Meyer FA, Hery C, Larochelle M. Women’s perceptions of events impeding or facilitating the detection, investigation and treatment of breast cancer. *Eur J Cancer Care (Engl)*. 2006;15(2):183–93. 10.1111/j.1365-2354.2005.00635.x. 16643266 10.1111/j.1365-2354.2005.00635.x

[22] Fayanju OM, Ren Y, Stashko I, et al. Patient-reported causes of distress predict disparities in time to evaluation and time to treatment after breast cancer diagnosis. *Cancer*. 2021;127(5):757–68. 10.1002/cncr.33310. 33175437 10.1002/cncr.33310 PMC7897266

[23] Blazek A, O’Donoghue C, Terranella S, et al. Impact of inequities on delay in breast cancer management in women undergoing second opinions. *J Surg Res*. 2021;268:445–51. 10.1016/j.jss.2021.06.084. 34416417 10.1016/j.jss.2021.06.084

[24] Golshan M, Losk K, Kadish S, et al. Understanding process-of-care delays in surgical treatment of breast cancer at a comprehensive cancer center. *Breast Cancer Res Treat*. 2014;148(1):125–33. 10.1007/s10549-014-3124-2. 25270121 10.1007/s10549-014-3124-2

[25] Mac Bride MB, Neal L, Dilaveri CA, et al. Factors associated with surgical decision making in women with early-stage breast cancer: a literature review. *J Womens Health (Larchmt)*. 2013;22(3):236–42. 10.1089/jwh.2012.3969. 23428286 10.1089/jwh.2012.3969

[26] Houssami N, Sainsbury R. Breast cancer: Multidisciplinary care and clinical outcomes. *Eur J Cancer*. 2006;42(15):2480–91. 10.1016/j.ejca.2006.05.023. 16904313 10.1016/j.ejca.2006.05.023

[27] Bhardwaj PV, Mason H, Kaufman SA, Visintainer P, Makari-Judson G. Outcomes of a multidisciplinary team in the management of patients with early-stage breast cancer undergoing neoadjuvant chemotherapy at a Community Cancer Center. *Curr Oncol*. 2023;30(5):4861–70. 10.3390/curroncol30050366. 37232824 10.3390/curroncol30050366 PMC10217230

[28] Tsai CH, Hsieh HF, Lai TW, Kung PT, Kuo WY, Tsai WC. Effect of multidisciplinary team care on the risk of recurrence in breast cancer patients: A national matched cohort study. *Breast*. 2020;53:68–76. 10.1016/j.breast.2020.07.001. 32652461 10.1016/j.breast.2020.07.001 PMC7375674

[29] Newman EA, Guest AB, Helvie MA, et al. Changes in surgical management resulting from case review at a breast cancer multidisciplinary tumor board. *Cancer*. 2006;107(10):2346–51. 10.1002/cncr.22266. 16998942 10.1002/cncr.22266

[30] Churilla TM, Egleston BL, Murphy CT, et al. Patterns of multidisciplinary care in the management of non-metastatic invasive breast cancer in the United States Medicare patient. *Breast Cancer Res Treat*. 2016;160(1):153–62. 10.1007/s10549-016-3982-x. 27640196 10.1007/s10549-016-3982-x PMC5064835

[31] American Cancer Society: About Breast Cancer. 25 Jan 2022

